# *Toxoplasma gondii* Relies on Both Host and Parasite Isoprenoids and Can Be Rendered Sensitive to Atorvastatin

**DOI:** 10.1371/journal.ppat.1003665

**Published:** 2013-10-17

**Authors:** Zhu-Hong Li, Srinivasan Ramakrishnan, Boris Striepen, Silvia N. J. Moreno

**Affiliations:** Center for Tropical and Emerging Global Diseases and Department of Cellular Biology, University of Georgia, Athens, Georgia, United States of America; University at Buffalo, United States of America

## Abstract

Intracellular pathogens have complex metabolic interactions with their host cells to ensure a steady supply of energy and anabolic building blocks for rapid growth. Here we use the obligate intracellular parasite *Toxoplasma gondii* to probe this interaction for isoprenoids, abundant lipidic compounds essential to many cellular processes including signaling, trafficking, energy metabolism, and protein translation. Synthesis of precursors for isoprenoids in Apicomplexa occurs in the apicoplast and is essential. To synthesize longer isoprenoids from these precursors, *T. gondii* expresses a bifunctional farnesyl diphosphate/geranylgeranyl diphosphate synthase (TgFPPS). In this work we construct and characterize *T. gondii null* mutants for this enzyme. Surprisingly, these mutants have only a mild growth phenotype and an isoprenoid composition similar to wild type parasites. However, when extracellular, the loss of the enzyme becomes phenotypically apparent. This strongly suggests that intracellular parasite salvage FPP and/or geranylgeranyl diphosphate (GGPP) from the host. We test this hypothesis using inhibitors of host cell isoprenoid synthesis. Mammals use the mevalonate pathway, which is susceptible to statins. We document strong synergy between statin treatment and pharmacological or genetic interference with the parasite isoprenoid pathway. Mice can be cured with atorvastatin (Lipitor) from a lethal infection with the TgFPPs mutant. We propose a double-hit strategy combining inhibitors of host and parasite pathways as a novel therapeutic approach against Apicomplexan parasites.

## Author Summary

*Toxoplasma gondii* is an obligate intracellular parasite and is not able to replicate outside the host cell. The parasite lives in a specialized parasitophorous vacuole in contact with the host cytoplasm through the parasitophorous vacuole membrane. It is highly likely that a very active exchange of metabolites occurs between parasite and host cell. We present evidence for this exchange for isoprenoids, abundant lipidic compounds essential to many cellular processes including signaling, trafficking, energy metabolism, and protein translation. Our work shows that intracellular *T. gondii* tachyzoites are able to salvage farnesyl diphosphate (FPP) and/or geranylgeranyl diphosphate (GGPP) from the host, and the parasite is able to grow even when its endogenous production is shut down. However, when extracellular, the parasite depends entirely on its own production of isoprenoids. We propose to use a combination of inhibitors that would hit both the host and the parasite pathways as a novel therapeutic approach against *Toxoplasma gondii* that could also work against other Apicomplexan parasites.

## Introduction

*Toxoplasma gondii* is an important intracellular pathogen causing disease in humans and animals. Most human infections are uncomplicated but the parasite persists and the chronic infection can be reactivated upon immunosuppression in patients undergoing organ transplants, cancer chemotherapy [1], or AIDS due to HIV infection [2]. During pregnancy, infection causes congenital toxoplasmosis with serious consequences to the fetus [3]. There is also growing concern about outbreaks of severe ocular disease due to *T. gondii* in immunocompetent patients [4]. The parasite masterfully manipulates its host cell to insure favorable conditions for its survival and replication. *T. gondii* infection results in differential regulation of a variety of host signaling and metabolic pathways [5]. Many of these host changes are still not completely understood but it is quite likely that such modification of host pathways is essential for parasite growth and survival.

Isoprenoids are lipid compounds with many important functions. The enzymes that synthesize and use isoprenoids are among the most important drug targets for the treatment of cardiovascular disease, osteoporosis and bone metastases and have shown promise as antimicrobials in a number of systems [6]. *T. gondii* lacks the mevalonate pathway for the synthesis of isoprenoid precursors that is used by mammals but harbors a prokaryotic-type 1-deoxy-D-xylulose-5-phosphate (DOXP) pathway in the apicoplast. This pathway generates isopentenyl diphosphate (IPP) and dimethyallyl diphosphate (DMAPP). We recently demonstrated that the DOXP pathway is essential in *T. gondii* [7]. Knockout of 1-hydroxy-2-methyl-2-(E)-butenyl 4-diphosphate reductase (LytB), which catalyzes the generation of IPP and DMAPP in the final step of the DOXP pathway, or of DOXP reductoisomerase (DOXPRI), which catalyzes the second step of the DOXP pathway, were both lethal [7]. We also characterized the key enzyme of downstream isoprenoid synthesis in *T. gondii*, farnesyl diphosphate synthase (TgFPPS) [8]. Interestingly, we found it to be a bifunctional enzyme that can catalyze the condensation of IPP with three allylic substrates: DMAPP, geranyl diphosphate (GPP), and farnesyl disphosphate (FPP). The enzyme thus generates not only 15-carbon FPP but also 20-carbon GGPP [8]. A bifunctional FPPS has also been described in *Plasmodium falciparum* [9]. TgFPPS is inhibited by long alkyl chain (lipophilic) bisphosphonates, which are among the most active inhibitors of human GGPPS [10], as well as by short chain bisphosphonates like risedronate (aminobisphosphonates), which preferentially inhibit human FPPS. *T. gondii* engineered to overexpress *TgFPPS* requires considerably higher levels of bisphosphonates to achieve growth inhibition supporting the idea that the *T. gondii* enzyme is a target of bisphosphonates [11].

In this work we report that drugs acting on the mevalonate pathway, like statins, are active *in vitro* and *in vivo* against *T. gondii*. This is surprising as the parasite lacks this pathway. With the use of *null* mutants for the *TgFPPS* (*Δfpps*) we demonstrate why the parasite is sensitive to these inhibitors. We also show that the parasite is able to salvage some isoprenoid intermediates from the host while depending on its own synthetic machinery for others. Our results reveal a metabolic exchange between host and parasite that quite likely also occurs in other intracellular pathogens like *Plasmodium* or *Cryptosporidium*. To take advantage of these findings we propose a double-hit strategy combining inhibitors of both host (statins) and parasite (bisphosphonates) pathways. This strategy will allow leveraging the extensive clinical experience gained with statins towards the treatment of infections and potentially adapt it to other intracellular parasites.

## Results

### TgFPPS is required for growth of *T. gondii* under stress but not essential under all circumstances

**Figure 1:**
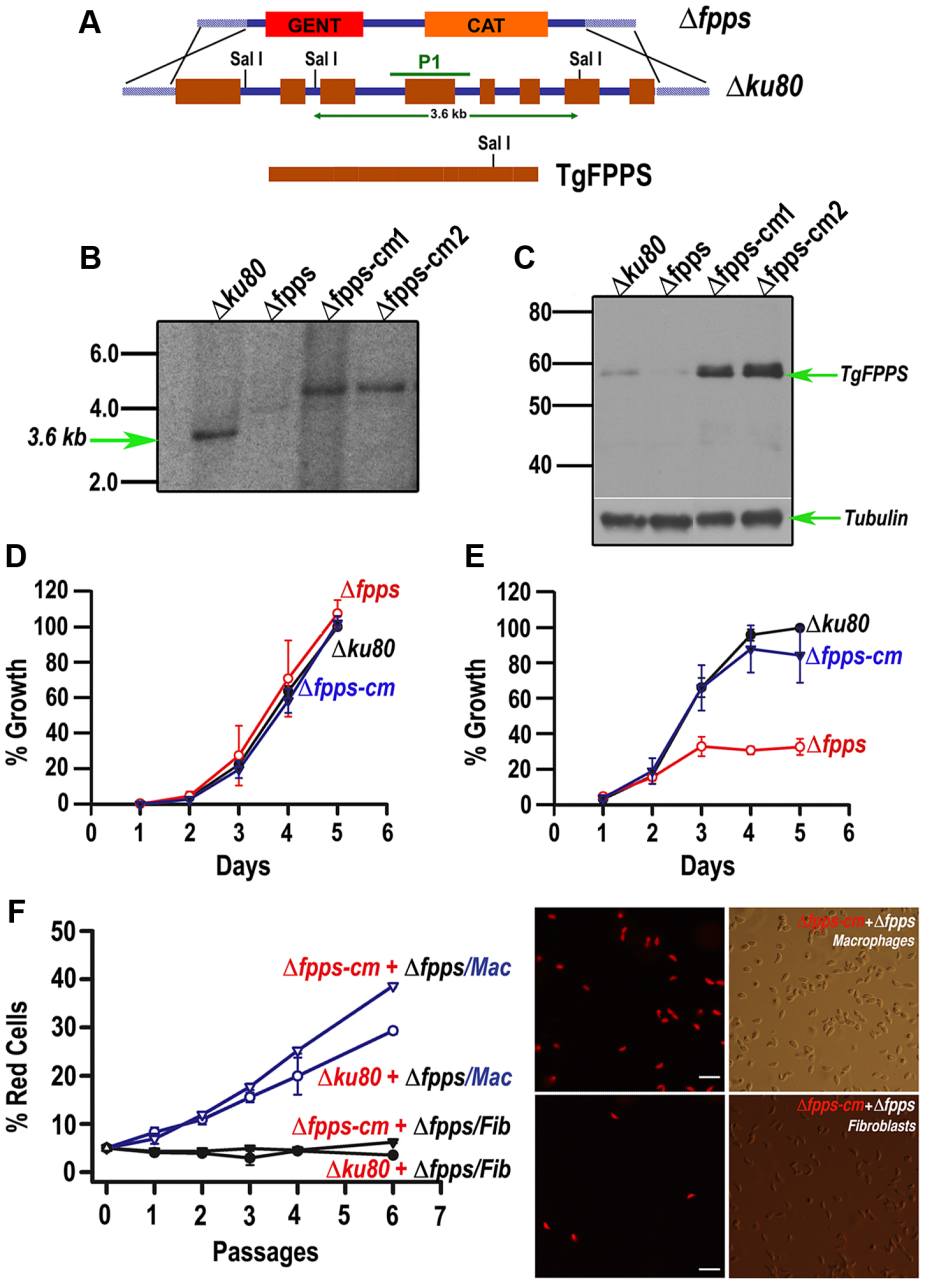
*TgFPPS* is essential for growth of *T. gondii* under stressful conditions. *A*, Cosmid strategy used to delete the *TgFPPS* gene. CAT: chloramphenicol acetyl transferase gene used for *T. gondii* selection. Restriction enzyme SalI cuts the *TgFPPS* genomic DNA three times and once the *TgFPPS* cDNA. P1 indicates the probe used for Southern blot analysis. ***B*** **,** Southern blot analysis with probe P1 shows deletion of the endogenous *TgFPPS* gene and its appearance in the complemented tachyzoites. *Δfpps*: *TgFPPS null* mutant, *Δfpps-cm1* and *Δfpps-cm2*: *TgFPPS null* mutant complemented with the *TgFPPS* gene (clone 1 and 2). ***C*** **,** Western blot analysis showing the absence of TgFPPS in the *Δfpps* mutants and its presence in the parental *Δku80*, *Δfpps-cm1* and *Δfpps-cm2* tachyzoites. ***D*** **,** Growth of *Δku80, Δfpps and Δfpps-cm (clone 1)* in fibroblasts followed by red fluorescence as in [7]. A standard curve was developed for fluorescence vs. number of parasites. ***E*** **,** Growth of *Δku80, Δfpps and Δfpps-cm* in macrophages J744. ***F*** **,** Growth competition assay in fibroblasts (*Fib, black*) or macrophages (*Mac, blue*). 5% of *Δku80* or *Δfpps-cm* (both of them expressing rfp) were mixed with 95% of *Δfpps* parasites. The fluorescence level at each passage follows growth of parental or complemented cells. Both, *Δku80* or *Δfpps-cm* can overgrow *Δfpps* parasites in macrophages (see increasing red fluorescence only in macrophages). ***G*** **,** Fluorescence microscopy of parental tachyzoites expressing tandem tomato RFP protein and grown in macrophages (*upper panels*) or fibroblasts (*lower panels*) showing their predominant growth in macrophages and only a small number of fluorescent parasites when grown in fibroblasts. Data in ***D***, ***E,*** ** ***F*** represent means ± SD of n = 3. The Y axis of ***D***, ***E*** and ***F*** indicates % parasites considering 100% the number of parasites of the *Δku80* strain at day 5.

FPPS is an essential component of the isoprenoid biosynthesis pathway in all cells studied so far. This enzyme synthesizes both FPP and GGPP in *T. gondii* and localizes to the mitochondria [8]. Previous work from our laboratory has shown that the *T. gondii* FPPS is inhibited by bisphosphonates, which also inhibit parasite growth. Considering the central role of this enzyme in the isoprenoid pathway we wanted to validate the entire pathway as potential target for chemotherapy. We approached this by creating a *null* mutant for the *TgFPPS* gene. We used the *T. gondii Δku80* strain, which favors homologous recombination [12], [13]. Our targeting construct was a large genomic cosmid recombineered to replace the gene with a drug resistance marker (Fig. 1A) [14]. After initial unsuccessful attempts, we were able to obtain *null* mutants when supplementing the medium with geranylgeraniol during the selection process. This requirement for geranylgeraniol for growth of mutant parasites is possibly because of their specific metabolite need during the stress of the transfection. We analyzed these mutant clones (*Δfpps*) by Southern (Fig. 1B) and western (Fig. 1C) blot and demonstrated the lack of both *TgFPPS* gene and protein. We isolated complemented clones by re-introducing the *TgFPPS* gene into the *T. gondii* genome (*Δfpps-cm1 and Δfpps-cm2*; Figs. 1B, and 1C). We next introduced tandem tomato red fluorescent protein constructs into all strains *(Δku80-rfp, Δfpps-rfp, Δfpps-cm-rfp)* to measure parasite growth following the intensity of red fluorescence [15]. To our surprise, *Δfpps* mutant parasites were able to grow at a similar rate in fibroblasts (the cells we routinely use for parasite culture, Fig. 1D), and formed plaques of similar number and size when compared to the parental and complemented strains (Figure S1, *top row*).

Previous work has shown that *T. gondii* can enter macrophages by active invasion. However weakened or stressed parasites can be actively phagocytized by macrophages, resulting in parasite death making macrophages a more challenging host cell than fibroblasts [16]. We tested our mutants for their ability to grow in macrophages. Interestingly, *Δfpps* parasites showed a significant growth defect in these cells (Fig. 1E, *red line*). We compared growth of our mutants in fibroblast vs. macrophages using a competition growth assay. For this, we mixed unlabeled *Δfpps* mutants with fluorescent parental (*Δku80-rfp*) or complemented strains *(Δfpps-cm-rfp)* at a 20∶1 starting ratio. Fig. 1F and 1G show that parental and complemented clones are able to rapidly outgrow mutant parasite in macrophages (*Mac*, *blue lines*) while they grow at a similar rate in fibroblasts (*Fib*, *black lines*). Our interpretation of these results is that whether the enzyme is required or dispensable for growth of the parasite depends on the specific host cell and host-parasite interaction. In this context we note that growth of *Δfpps* mutants was well supported in low passage primary fibroblasts, as measured by plaque assay (Fig. S1, *top row*), but limited in aging fibroblast cultures (fibroblast with ∼40 passages; Fig. S1, *bottom row*). This suggests that the parasite isoprenoid metabolism may not only be sensitive to the cell type infected but also to its physiological and/or metabolic state.

We next addressed whether *Δfpps* mutants would be less virulent *in vivo*. The RH strain is hypervirulent, which can make it difficult to appreciate modest attenuation. We observed a difference in virulence when infecting mice with low parasite numbers (5–10) while higher doses (15–100) lead to death at 9–10 days (data not shown). We wondered whether the use of a less virulent strain would better highlight the difference in virulence between mutant and parental cell lines. We created conditional mutants using the described TATi cell lines [17]. There are two advantages for using these cells. First, the reduced virulence of the parental cell line allows the use of 10^4^–10^5^ parasites to infect mice. Second, these mutants are maintained in culture expressing an extra copy of *TgFPPS*, prior to suppression with anhydrotetracycline (ATc) thus avoiding preadaptation. We first expressed a regulatable copy of the TgFPPS in the TATi parental cell line (Fig. S2A) and created the cell line FPPS/FPPSi. In a clonal line derived from these cells we disrupted the endogenous *TgFPPS* gene as detailed before (see legend for Fig. S2) and generated ΔFPPS/FPPSi mutants. We established ATc regulation and gene deletion by western and Southern blot analyses, respectively (Fig. S2B and S2C). Plaque assays in fibroblasts in the presence of ATc showed no difference in the number and size of plaques (Fig. S2D). In contrast, a highly significant difference in growth was observed when parasites were allowed to infect macrophages (Fig. S2E), as seen before with the *Δfpps* mutants (Fig. 1E) indicating a fitness defect only evident under stressful conditions.

With the purpose to define a dependable inoculum to use for virulence studies, we first established a protocol in which we passed our Tati-derived strains through mice and performed *in vivo* titration experiments (see Materials and Methods and Fig. S3). This treatment increased consistency dramatically and we found that using an inoculum defined in this way resulted in reproducible virulence outcomes. To establish whether FPPS knockdown affects the ability of these parasites to cause disease, mice were infected with 10,000 FPPS/FPPSi or ΔFPPS/FPPSi tachyzoites (Fig. S3) (a reproducible number found after our *in vivo* titration experiments). Mice challenged with the FPPS/FPPSi strain succumbed to the infection even if they were given ATc in the water (Fig. S3, *black lines*). In contrast, mice infected with the ΔFPPS/FPPSi and receiving ATc survived the infection while mice infected with the same parasites but given a placebo were susceptible to infection (Fig. S3, compare *red lines*).

### TgFPPS knockout parasites have stress-related phenotype

**Figure 2:**
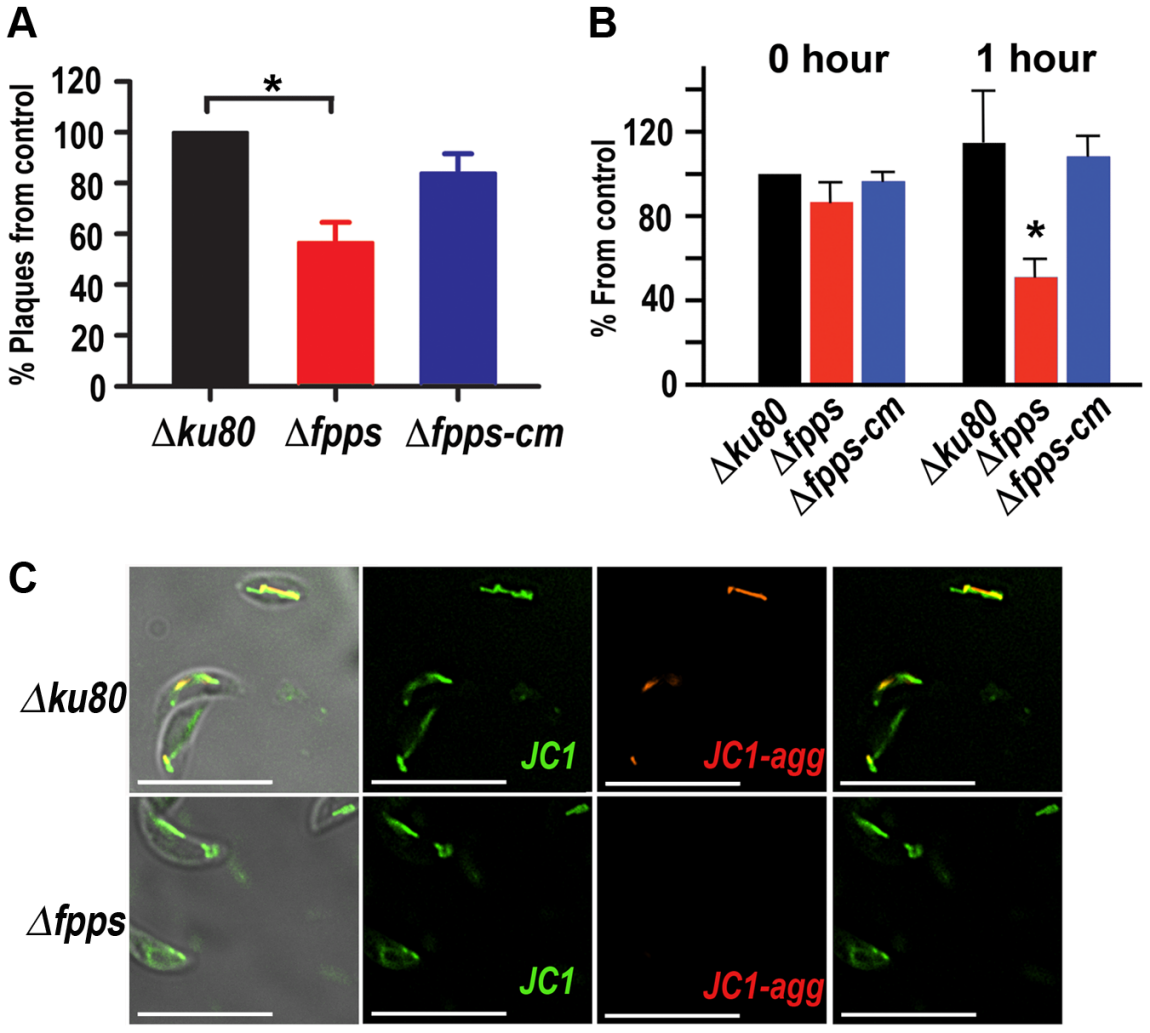
*TgFPPS* knockout parasites have a mitochondrial defect. *A*, Freshly egressed parasites were filtered, collected, and resuspended in DMEM medium. These parasites were incubated in DMEM medium at 37°C for 0.5 hs before allowing them to infect hTERT fibroblasts. Plaques were counted as detailed under Materials and Methods. The data plotted are from 3 independent experiments. The number of plaques for the *Δfpps* and for the *Δfpps-cm* are normalized considering the number of plaques for the parental cells *Δku80* as 100% (*Δfpps vs Δku80, P = 0.002, *Δfpps vs Δfpps-cm: P = 0.08, not significant) ***B*** **,** ATP levels of recently released parasites (0 hour) or parasites incubated in buffer simulating extracellular ionic conditions for one hour (1 hour). The Y-axis indicates % ATP level considering the value obtained from the *Δku80* strain at time 0 as 100%. Results are expressed as means ± S.D. of n = 3. Other experimental conditions are under Materials and Methods. (At 1 hour: *Δfpps vs Δku80, P = 0.002, *Δfpps vs Δfpps-cm: P = 0.0004) ***C*** **,** Fluorescence analysis of *Δku80* and *Δfpps* tachyzoites stained with JC1, showing decrease in mitochondrial membrane potential of *Δfpps* tachyzoites (note the decrease in red fluorescence).

With the aim of understanding how the *Δfpps* mutant parasites manage to survive without the production of essential isoprenoids we measured growth of mutant *Δfpps* parasites and their parental strain after being deprived of host cells for a determined length of time. We exposed mutant, parental and complemented parasites for 30 min to an extracellular buffer and for a more accurate readout we switched to a plaquing efficiency protocol as described [18] in which there is only a 30-min contact interval between parasite and host (Figure 2A). Plaques were counted after 7 days of incubation. We observed that the number of plaques was significantly lower for the *Δfpps* parasites after being exposed to these stress conditions. We also measured ATP levels of parasites incubated in extracellular media for one hour. No difference in the ATP levels was observed in recently egressed parasites but there was a significant decrease in the *Δfpps* mutants after incubating them for one hour in extracellular buffer with glucose (Figure 2B). These results indicate that the *Δfpps* parasites do have a defect in energy generation, which is not evident under the protected intracellular environment. However, this defect becomes relevant when the parasite is outside the host and we were able to increase it by incubating them for an extended length of time before letting them continue with its lytic cycle (Figure 2A). A possible cause of this defect could be the synthesis of ubiquinone, isoprenylated cofactor of the mitochondrial respiratory chain, which may be more important when the parasites are extracellular. We measured mitochondrial membrane potential of *Δfpps* and the parental *Δku80* parasites using JC1, a lipophilic, cationic dye that accumulates in mitochondria in a membrane potential dependent fashion and that changes color from green to red as it accumulates [14] (Figure 2C, *upper panels*). The lower panels show that for the mutant parasites JC1 stays mostly green indicative of a partially depolarized mitochondrial membrane (Figure 2C, *lower panels*). We also used flow cytometry to quantify the effect of knocking out the *TgFPPS* gene (*Δfpps*) and compare it with the parental strain *Δku80* (Figure S4). We observed a dramatic drop of mitochondrial fluorescence in *Δfpps* parasites (56.2%, compared to 85.2%). This indicates a mitochondrial defect that is not important for intracellular life but becomes accentuated when the parasites are deprived of host cells.

### *T. gondii* can salvage isoprenoid intermediates from its host

**Figure 3:**
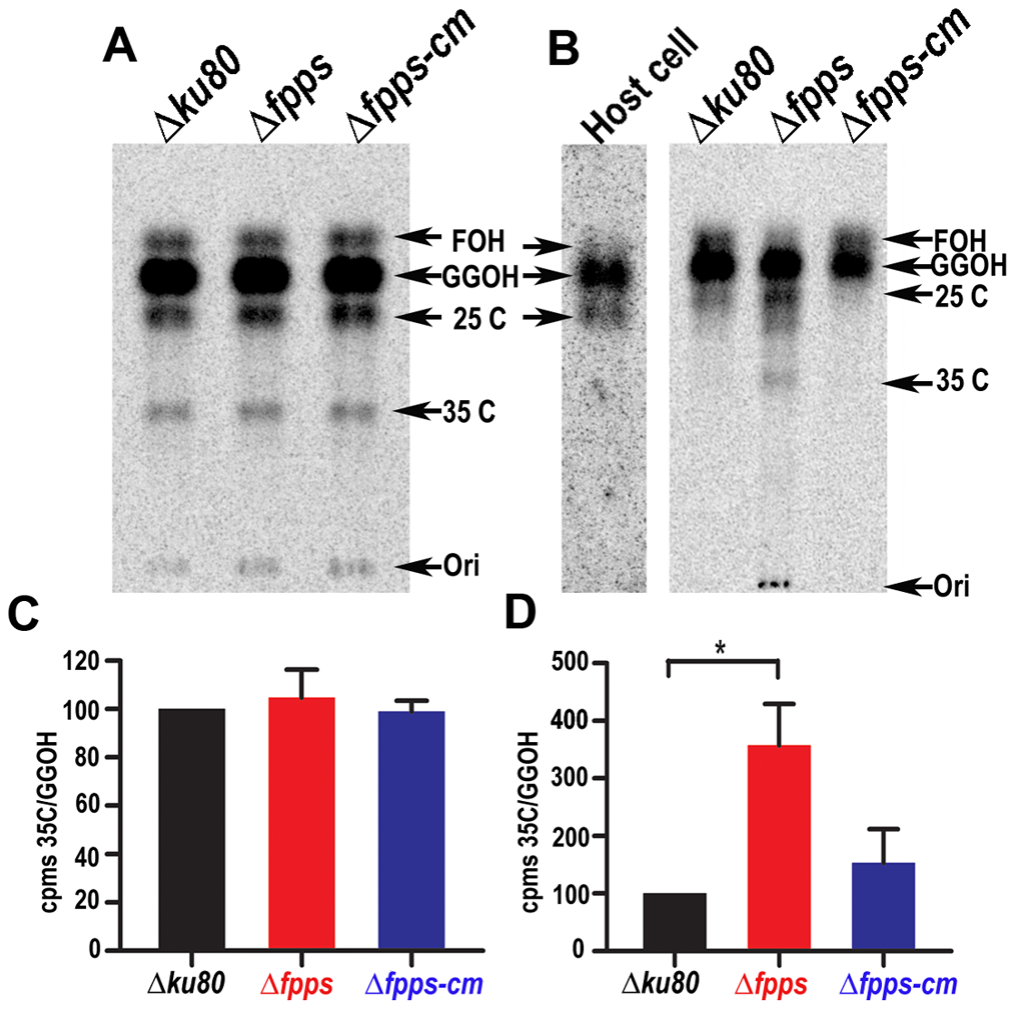
*T. gondii* can salvage isoprenoid intermediates from its host. *A*, *T. gondii*-infected fibroblasts were labeled with ^14^C -glucose. Isoprenoids were extracted from purified tachyzoites and analyzed by thin layer chromatography (TLC); ***B*** **,** Fibroblasts were labeled with ^14^C-glucose for 2 days and infected with *T. gondii* after washing with non-radiolabeled medium. Tachyzoites were collected after growing without ^14^C-glucose and purified for further extraction of their isoprenoids as described under Materials and Methods. Isoprenoids were hydrolyzed to their corresponding alcohols and analyzed by TLC using a previously described system [8]. Ori indicates the origin, FOH, farnesol, GGOH, geranylgeraniol, 25C and 35C are isoprenoid products that run in this system as isoprenoids with that number of carbons. The length of the isoprenoid products was calculated from a standard curve made using known isoprenoid standards and measuring the length of the run for each compound. This a is representative TLC from more than 3 independent experiments with similar results. ***C*** **,** Calculation of the ratio between the cpm obtained for the 35C and GGOH spots obtained in ***A*** **,** using a phosphoroimager and multiplied by 100. ***D*** **,** Same as in C for the TLC in ***B***.

Our surprising findings could imply that intracellular parasites salvage isoprenoids from their host and that we impinge on this ability by cultivation in different cells. To test this hypothesis, we performed two labeling experiments testing different conditions. We first labeled infected fibroblasts with ^14^C-glucose (Figure 3A). Under these conditions, radioactive glucose is available to both host and parasite to label isoprenoids generated by host and parasite specific *de novo* synthesis pathways. In the second experiment the strategy was to first label the fibroblasts with ^14^C-glucose, remove unincorporated label by washing the cells with fresh medium and only then infect with parasites (Figure 3B). In both settings we compared parental (*Δku80*), mutant *(Δfpps)*, or complemented (*Δfpps-cm*) *T. gondii*. Parasites were purified through several filtration steps before isoprenoid extraction.

Parasite isoprenoids were isolated by solvent extraction of purified tachyzoites and analyzed by thin layer chromatography and autoradiography (TLC). When infected cells were labeled (Figure 3A) the most abundant isoprenoids were FPP, GGPP, an intermediate co-migrating with a 25C standard (25 C), and a longer unidentified derivative co-migrating with a 35C standard (long prenyl diphosphate; LPP, 35C). The results indicate that mutant parasites (*Δfpps*) have levels of intermediates similar to the parental strain despite the lack of FPPS (differences between labeled compounds were not statistically significant, n = 3, data not shown). Figure 3B shows the isoprenoids obtained from the parasite after labeling only the host cells followed by infection with unlabeled parasites. FPP and GGPP were still present and labeled in the extracts obtained from mutant parasites despite the fact that they lack the enzyme required for their synthesis (Figure 3B, *Δfpps*). However, labeling of the longer chain product was stronger in extracts from *Δfpps* mutants. This likely indicates that the parasite synthesizes these longer chain products using both host and its own precursors, and that labeling via the host pathway becomes more evident in the absence of parasite synthesis (Figure 3B, *Δfpps*). The parental and complemented cells did not show this labeling arguing that it is generated in the parasite using unlabeled precursors. The results were quantified by calculating the ratio of labeling for this long chain product to that of GGOH and is displayed with bar graphs in Figs. 3C and 3D. This analysis shows no difference between mutant and wild type when parasite and host cells are simultaneously labeled with ^14^C-glucose (Figure 3C). However, the ratio was significantly higher for the mutant parasite when only the host cells were prelabeled (Fig. 3D). Taken together these results suggest that mutant parasites lacking their own production of FPP and GGPP import these intermediates from the host (pre-labeled with ^14^C in our experimental set-up) and convert them into the long chain isoprenoid. Under similar experimental conditions, when analyzing the isoprenoid products made by the parental strain, the labeling of this long chain isoprenoid product becomes diluted as a consequence of the endogenous production of unlabeled FPP and GGPP by the TgFPPS.

### Inhibition of the host mevalonate pathway enhances the requirement for parasite isoprenoid synthesis

**Figure 4:**
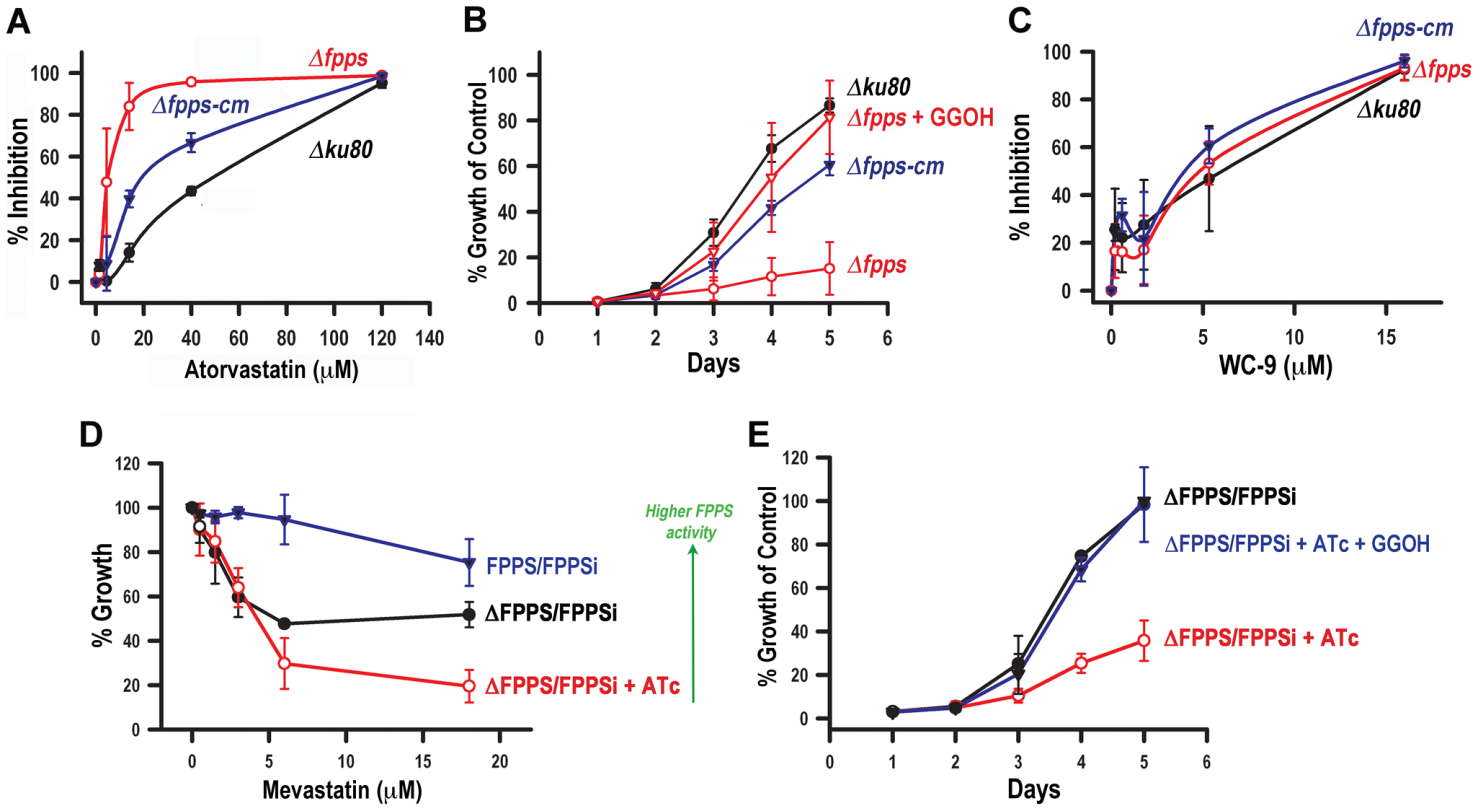
Inhibition of the host and parasite isoprenoid pathways. *A*, Growth inhibition of *Δku80*, *Δfpps* and *Δfpps-cm* tachyzoites by atorvastatin. ***B*** **,** *Δku80*, *Δfpps* and *Δfpps-cm* tachyzoites were cultured in the presence of 13 µM atorvastatin. Where indicated (*Δfpps*+GGOH) geranylgeraniol (1 µM) was added to the culture medium. ***C*** **,** Growth inhibition of *Δku80*, *Δfpps* and *Δfpps-cm* tachyzoites by the squalene synthase inhibitor WC-9. ***D*** **,** Growth inhibition of the *T. gondii* FPPS/FPPSi, ΔFPPS/FPPSi and ΔFPPS/FPPSi+ATc in the presence of different concentrations of mevastatin. ***E*** **,** FPPS/FPPSi, ΔFPPS/FPPSi and ΔFPPS/FPPSi+ATc were cultured in the presence of 5 µM mevastatin. Where indicated (ΔFPPS/FPPSi+ATc+GGOH) geranylgeraniol (1 µM) was added to the culture medium. The number of parasites at day 5 in the absence of inhibitor is considered as 100%. Results are expressed as means ± SD of n = 3.

If the parasite is taking up FPP and/or GGPP from the host, then inhibiting the synthesis of these host compounds may affect parasite growth. We directly tested this idea using an inhibitor of hydroxymethyl glutaryl-CoA reductase (HMG-CoA reductase), the rate-limiting enzyme of the host mevalonate pathway (this pathway is absent in *T. gondii*). We tested atorvastatin (Lipitor) in tissue cultures (Fig. 4A and 4B, *black lines*) and found that atorvastatin is able to inhibit growth of the parental strains with an IC_50_ of ∼40 µM. We thought that this modest level of efficacy points to other sources of FPP and GGPP for the parasite, in particular its own synthesis. We hypothesized that the *Δfpps* mutants, unable to produce FPP and GGPP should be more sensitive to the inhibition of the host by atorvastatin. This is indeed what we observed when testing the drug against the mutant parasites (Fig. 4A and B, *red lines*) and we calculated an IC_50_ of 2 µM (20 times lower than the efficacy against the parental cell lines). This effect of atorvastatin is specific to its inhibition on the production of isoprenoid metabolites because it was possible to rescue parasite growth by adding geranylgeraniol to the medium (Fig. 4B, *red lines: Δfpps* and *Δfpps+GGOH*).

To investigate whether atorvastatin inhibits parasite growth mainly as a result of impaired cholesterol synthesis we tested WC-9, a known inhibitor of squalene synthase (SQS) [19]. We found WC-9 to inhibit parasite growth with an IC_50_ of 4–5 µM (Fig. 4C). *T. gondii* does not encode SQS and acquires cholesterol from its host [20], [21], [22]. We therefore attributed the effect of WC-9 to its action against the host pathway. Importantly, we found no difference in the WC-9 susceptibility of *Δku80, Δfpps* and *Δfpps-cm* parasites (Fig. 4C). This suggests that WC-9 acts downstream of the formation of FPP and GGPP, and that inhibition of cholesterol synthesis is not the most important anti-parasitic effect of statin treatment. This is in agreement with previous findings that suggested that the parasite relies on LDL trafficking rather than de novo synthesis by the host cell to satisfy its cholesterol requirement [20], [21], [22].

We next tested another statin (mevastatin) on FPPS/FPPSi or ΔFPPS/FPPSi parasites grown in the presence or absence of ATc (Figs. 4D). FPPS/FPPSi tachyzoites express an extra copy of *TgFPPS* (Fig. S2B) and possesses higher FPPS activity (not shown). There was a reverse correlation between mevastatin inhibition and the expression level of *TgFPPS* (Fig. 4D). ΔFPPS/FPPSi parasites were the most susceptible in the presence of ATc (IC_50_∼4 µM mevastatin) while FPPS/FPPSi cells with an extra copy of the *TgFPPS* gene were resistant to concentrations up to 18 µM (Fig. 4D). The effect of mevastatin was rescued by supplementation of the medium with 1 µM geranylgeranyol (Fig. 4E, compare *red* and *blue lines*), again supporting its direct effect on the production of FPP and GGPP.

**Figure 5:**
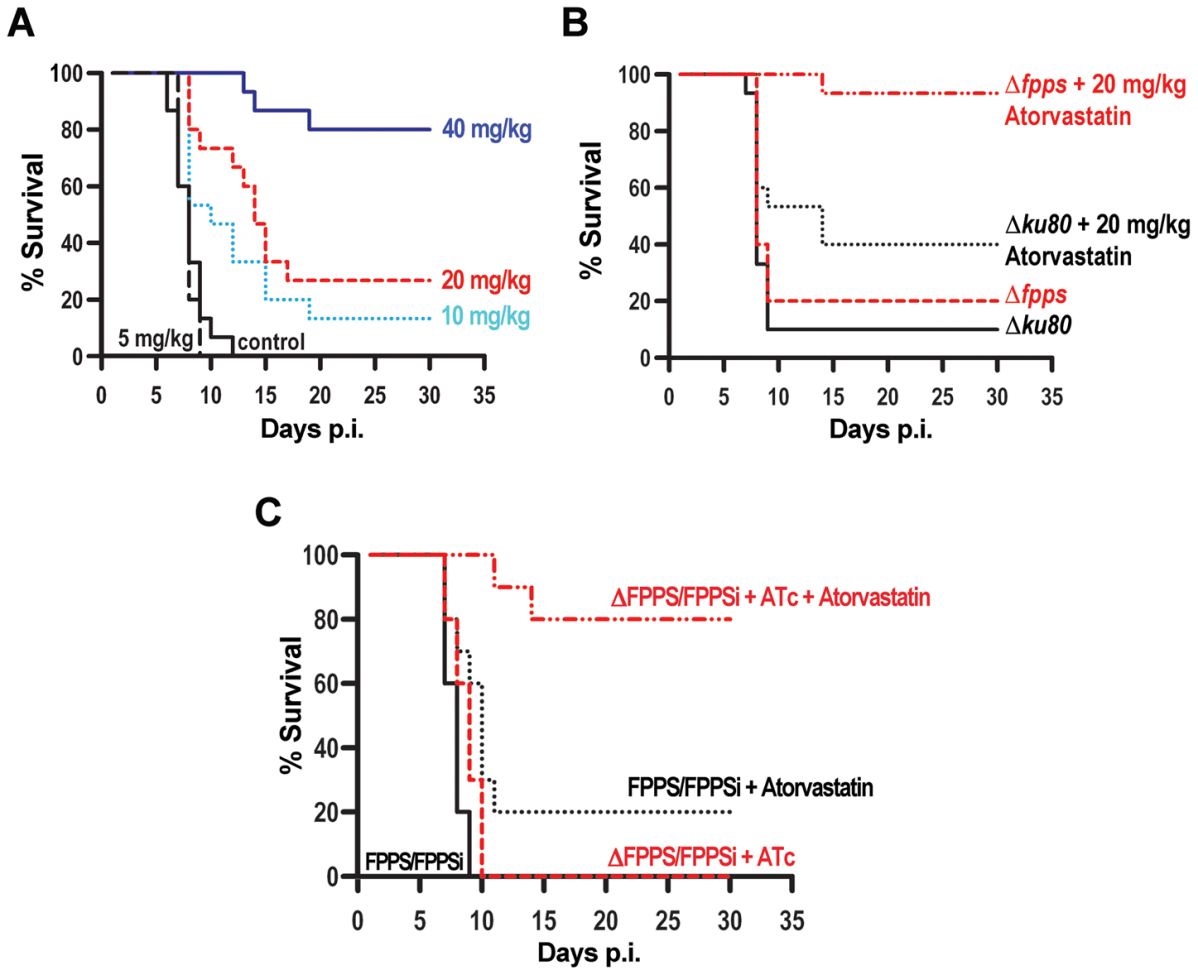
Effect of atorvastatin in mice infected with *T. gondii.* ***A*** **,** Atorvastatin treatment of mice infected with the RH strain. 20 RH tachyzoites were injected i.p. into Swiss Webster mice. Atorvastatin was given i.p. daily starting 6 hours after infection for 15 days. Results are the summary of 4 independent experiments. ***B*** **,** Atorvastatin can cure mice infected with a lethal dose of *Δfpps* parasites. 10 *Δku80* or *Δfpps* tachyzoites were inoculated (i.p.) into Swiss Webster mouse. Atorvastatin (20 mg/kg/day) started 6 hours after infection for 10 days. Results are from 3 independent experiments using 10 mice for each group. ***C*** **,** Atorvastatin cures a lethal infection with ΔFPPS/FPPSi parasites. Balb/c mice were infected with 100,000 fresh tachyzoites (i.p.) of FPPS/FPPSi (*black lines*) or ΔFPPS/FPPSi (*red lines*). Atorvastatin protect mice against death if the infection with ΔFPPS/FPPSi is followed by the administration of 0.2 µg/ml anhydrotetracyclin ATc to suppress the expression of the extra copy of TgFPPS (*red dashed line*) (80% mice survive). Only 20% of mice infected with FPPS/FPPSi parasites survive if treated with atorvastatin (*black dotted line*). The results shown are from 2 independent experiments (10 mice each group). Treatment with atorvastatin (20 mg/kg×day, i.p.) was started 6 hours after infection for 10 days for the groups indicated. Note that graphs start at day 1 but infection is done on day 0.

We also tested the efficacy of atorvastatin treatment against *T. gondii* infection of mice using wild type RH strain. Fig. 5A shows a summary of 3 experiments using groups of 5 mice treated with different doses of atorvastatin. While 100% of control mice died between 9–13 days post-infection, 80% of mice treated with the higher 40 mg/kg/day dose, survived more than 30 days. Note that this is not an excessive drug dose but the standard concentration of atorvastatin commonly used and well tolerated in mouse experiments [23], [24]. An atorvastatin ED_50_ of 32.3 mg/kg per day was calculated (Fig. 5A). We also were interested in comparing the efficacy of atorvastatin against the infection of mice with *Δku80*, and *Δfpps* cells. We infected mice with a lethal dose of parasites (parental and mutants) to highlight the effect of atorvastatin against infection with the *Δfpps* clone. Fig. 5B shows that atorvastatin is highly effective at treating mice infected with *Δfpps* parasites: 9 of 10 mice survived the infection when treated with atorvastatin, while 8 of 10 mice died in the absence of atorvastatin. To establish further that knockdown of *TgFPPS* make *T. gondii* infection more amenable to treatment with atorvastatin we infected mice with a lethal dose of 100,000 ΔFPPS/FPPSi or FPPS/FPPSi tachyzoites and treated with ATc in their drinking water (Fig. 5C). This high parasite dose was lethal even when infecting with ΔFPPS/FPPSi (compare with Fig. S3 for which we used 10,000 parasites, ten fold difference in dose) [25]. Most mice infected with FPPS/FPPSi and treated with atorvastatin succumbed to this high infection (Fig. 5C, *black lines*). In contrasts most mice infected with ΔFPPS/FPPSi were cured by atorvastatin when the mutation was induced by ATc treatment (Fig. 5C, *red lines*).

### *In vitro* drug interaction of atorvastatin and parasite isoprenoid pathway inhibitors

**Figure 6:**
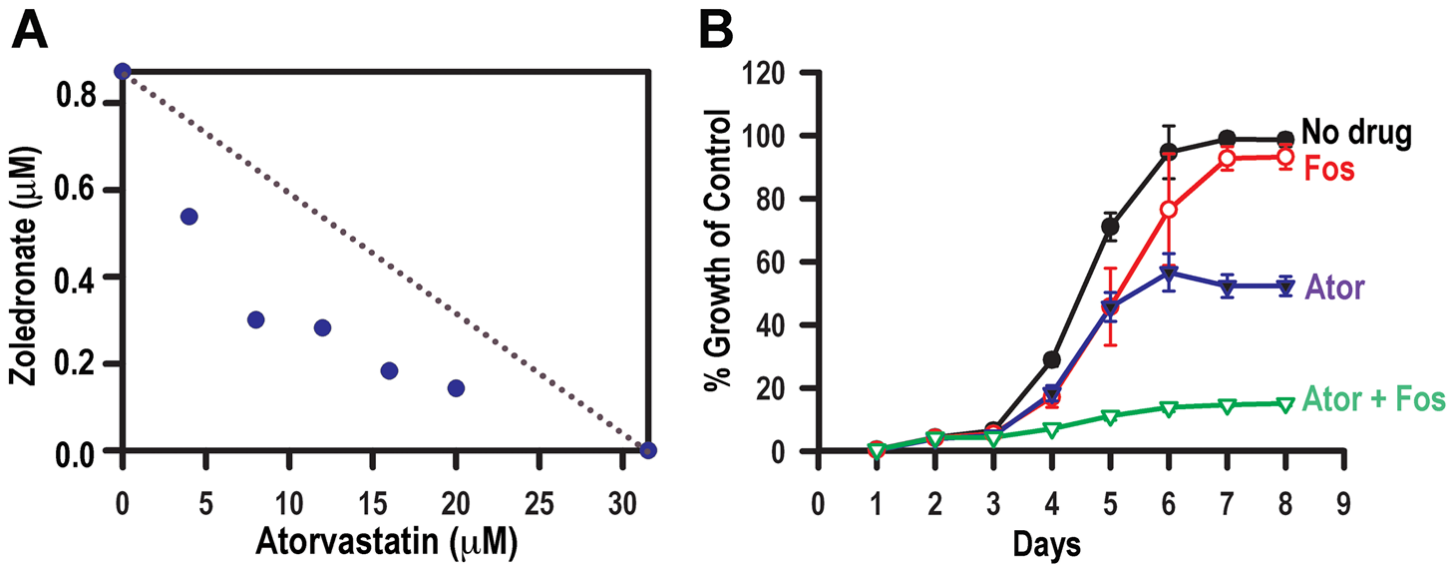
Combination of atorvastatin with an inhibitor of the TgFPPS and with an inhibitor of the DOXP pathway. *A*, Isobolograms of the interaction of atorvastatin and zoledronate. Axes are all normalized IC_50_s. Data are from 3 independent experiments. The dotted line indicates the hypothetical additive curve. ***B*** **,** Growth curve of tachyzoites expressing GlpT in the presence of 50 µM atorvastatin and 0.78 µM fosmidomycin. Results are expressed as means ± S.D. of n = 3.

*T. gondii* appears to be able to rely on both synthesis and salvage of isoprenoids. *Δfpps* mutants are more dependent on salvage. Could this be exploited pharmacologically by combining inhibitors of TgFPPS with atorvastatin? Bisphosphonates are known inhibitors of FPPS and have shown antiparasitic activity [11]. We chose to test zoledronic acid [26] because our previous work had identified this compound as the bisphosphonate with the highest specificity against TgFPPS, and its activity decreased significantly when we overexpressed the parasite enzyme [11]. To evaluate interaction between atorvastatin and zoledronate, we mixed both drugs at different concentrations following a protocol designed for testing synergy [27]. This protocol measures and calculates the IC_50_ of one drug in the presence of subtherapeutic concentrations of the second drug [27] . The results were plotted in an inhibition isobologram using IC_50_s of individual drugs and of five different drug combinations (Fig. 6A). The resulting curve is concave for atorvastatin and zoledronic acid and thus indicative of synergistic drug interaction (Fig. 6A).

FPP and GGPP production in the parasite requires the isoprenoid precursors IPP and DMAPP. We therefore next wanted to test whether atorvastatin would interact with fosmidomycin, a specific inhibitor of the DOXP pathway. *T. gondii* is insensitive to fosmidomycin because the drug is not able to cross the parasite membrane [7]. However a *T. gondii* transgenic parasite that expresses the bacterial transporter glycerol-3-phosphate transporter (GlpT) capable of importing fosmidomycin, is sensitive to fosmidomycin [7] (Fig. 6B). We assessed the growth of these parasites in the presence of 50 µM atorvastatin and 0.78 µM of fosmidomycin. This represents the IC_10_ for fosmidomycin and this low concentration was deliberately chosen to be able to detect drug interaction. Individually these drugs affected parasite growth as expected, approximately 50% inhibition with 50 µM atorvastatin and very little inhibition with 0.78 µM fosmidomycin. Interestingly, combining both drugs abolished parasite growth, indicating strong interaction also between atorvastatin and fosmidomycin. We tested the interaction between atorvastatin and fosmidomycin in these transgenic parasites by a simplified checkerboard technique [27] and calculated the fractional inhibitory concentration (FIC) index to be 0.36, confirming synergistic interaction (FIC<0.5) [28], [29]. This assay provided additional strong evidence that the parasite, although capable of generating its own isoprenoids, also depends on the host isoprenoids for continuous growth and successful infection. Our results show that therapeutic strategies aimed at interfering with both parasite and host isoprenoid synthesis could provide a higher rate of success in curing *T. gondii* infections.

## Discussion

Our work reveals a crucial metabolic interaction between the intracellular pathogen *T. gondii* and its host cell to secure the parasite's access to isoprenoids. Isoprenoids are essential for all cells and in most Apicomplexans their five carbon precursors are produced by the apicoplast [30], [31]. The synthesis of these precursors is now viewed as the most important function of the apicoplast and the reason the organelle was maintained long after the loss of photosynthesis [32]. Genetic analysis in *T. gondii* demonstrates that loss of the apicoplast isoprenoid pathway is lethal and mimics complete loss of apicoplast metabolism [7], [14]. Inhibiting this pathway with the antibiotic fosmidomycin is effective against *Plasmodium*, *Babesia*, and against *Toxoplasma* (once parasites are engineered to take up the drug) [7], [33], [34]. Most intriguingly, in *Plasmodium falciparum* cells cured of their apicoplasts by antibiotic treatment targeting plastid translation can nonetheless be continuously maintained in culture when the media are supplemented with high concentrations of IPP [35]. Overall these studies suggest that the synthesis of IPP and DMAPP by the parasite is essential and cannot be circumvented by salvage from the host under physiological conditions.

**Figure 7:**
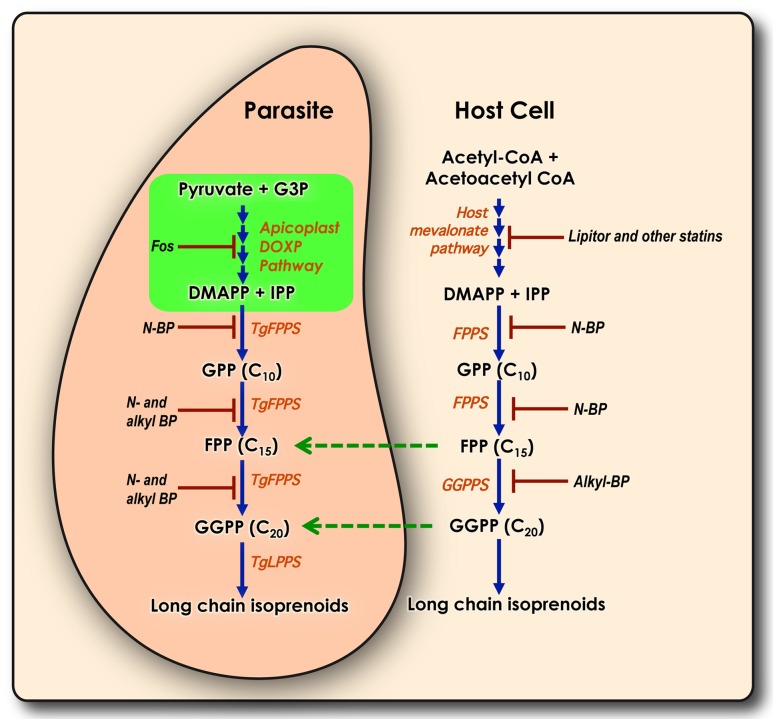
*Toxoplasma gondii* and host isoprenoid intermediates. The cartoon shows isoprenoid metabolites synthesized by *Toxoplasma gondii* tachyzoites (pink) and host (beige) and their interaction. The DOXP pathway enzymes localize to the parasite apicoplast (green). The mevalonate pathway enzymes are only present in the host. Green arrows show metabolites imported from the host as demonstrated in this work. The parasite enzyme TgFPPS synthesizes both FPP and GGPP while the host uses two enzymes (FPPS and GGPPS) to make the same metabolites. Enzymes and their known inhibitors are indicated. G3P: glyceraldehyde 3-phosphate; DMAPP: dimethyl allyl diphosphate; IPP: isopentenyl diphosphate; GPP: geranyl diphosphate; FPP: farnesyl diphosphate; GGPP: geranylgeranyl diphosphate; N-BP: nitrogen bisphosphonates; Alkyl-BP: alkyl bisphosphonates; Fos: fosmidomycin.

This makes our observation that the parasite enzyme catalyzing the next step in the isoprenoid pathway – the synthesis of FPP and GGPP from IPP and DMAPP is dispensable for *T. gondii* in fibroblasts all the more surprising. FPPS-catalyzed reactions are essential in most organisms studied so far and are important drug targets. *T. gondii* is not only able to make its own isoprenoids but can also import from the host cell (Fig. 7). We note that this ability to salvage appears not to be universal but restricted to certain compounds (Fig. 7). At the moment it is not fully understood whether this difference is due to difference in transport capability of the parasite or availability and abundance of the metabolites in the host cell. However, in our experiments we measured a strong impact of the host cell environment for FPP and GGPP. Extracellular parasites or parasites infecting macrophages rather than fibroblasts show more pronounced defects upon loss of the synthesis capacity. The intracellular survival of *T. gondii* depends on its unique ability to invade cells actively. Active invasion is fundamentally different from phagocytosis and requires parasite motility [16]. When extracellular parasites are incubated in PBS, their ability to invade cells actively rapidly declines, and they are mostly internalized by phagocytosis with parasites engulfed in phagosomes, which fuse with endosome/lysosomes and are further digested [16]. Parasite fitness is essential for its ability to actively invade host cells and/or escape from the phagosome. Lack of endogenous production of FPP and GGPP by *T. gondii* renders them less able to grow in macrophages. This could be the result of a fitness defect or because of a shortage of metabolites in macrophages or a different mechanism of transport of isoprenoids in these cells.

How dependent is the parasite on isoprenoid salvage under normal conditions with its synthesis capability intact? Our labeling studies show robust import of host cell-synthesized isoprenoids even in wild type parasites. Import is also supported indirectly by microarray studies of *T. gondii*-infected fibroblasts that revealed a significant induction of genes encoding enzymes of the mevalonate pathway following infection [5], [36] including the rate-limiting enzyme *HMG-CoA* reductase, and *FPPS* [5]. Previous work has shown that *T. gondii* does not synthesize cholesterol and imports it from the host low–density lipoprotein (LDL) [20], [21], [22]. It is possible that the inhibition of cholesterol synthesis by statins results in reduced parasite invasion or reduced parasite growth. Interestingly, a recent study has shown that atorvastatin treatment of endothelial cells reduced cytoadherence of *Plasmodium falciparum* [37].

We consider that inhibition of host cholesterol synthesis is unlikely as the reason for the effect of statins because of three reasons. First, the isoprenoid intermediate geranylgeraniol was able to rescue almost completely the growth inhibition by two statins, atorvastatin and mevastatin. Second, growth inhibition by an SQS inhibitor that blocks the pathway downstream to the production of FPP and GGPP was not enhanced in the *Δfpps* mutants, as observed with atorvastatin and mevastatin. And third, statins do not reduce overall plasma cholesterol levels in mice as they do in humans (due to low levels of low density lipoproteins in rodents) [38], [39]. In addition, it has been demonstrated that host cell cholesterol production has no significant effect on parasite replication and that the bulk of parasite cholesterol requirement can be satisfied by exogenous cholesterol from low-density-lipoprotein delivered to the parasitophorous vacuole [20]. Our results with the squalene synthase inhibitor strongly support that conclusion. It is interesting to note that the growth in macrophages of *Salmonella enterica* serovar Typhimurium, which also lacks a mevalonate pathway, is inhibited by statins and this inhibition is not due to a deficient production of sterols but of intermediates of the pathway between mevalonate and squalene 2,3-oxide [38]. It would be very interesting to investigate whether these intermediates are also FPP and GGPP as in the case of *T. gondii*.

Our labeling results indicate that *T. gondii* may use its own enzymes to make specific long chain isoprenoids. We previously reported that TgFPPS localizes to the mitochondria [8]. Our results showed that loss of TgFPPS resulted in alteration of the mitochondrial membrane potential, and rapid decrease in the ATP levels of extracellular parasites. These results suggest that TgFPPS functions to make FPP and GGPP in the mitochondrion as precursors for long chain isoprenoids and ubiquinone synthesis. Therefore we could deduce that TgFPPS plays an important role in maintaining mitochondrial function. This appears to be crucial for the parasite during its extracellular stage. Our results suggest a requirement for oxidative phosphorylation for generation of ATP in extracellular parasites. This is consistent with our previous results showing active oxidative phosphorylation in extracellular parasites [40] and a recent report showing that oxidative phosphorylation is responsible for >90% ATP synthesis in extracellular tachyzoites [41]. The deficient synthesis of ubiquinone precursors would not affect tachyzoites when they are intracellular while seriously impeding extracellular parasites as a consequence of rapid depletion of ATP, which is needed for gliding motility and invasion. Taken together, we show that *T. gondii* has a versatile system for its isoprenoid needs. During its replicative stage with needs for large quantities of isoprenoids, the parasite is able to manipulate the host and salvage isoprenoids. However, under stressful situations the parasite is able to provide by itself and this was emphasized when exposing *Δfpps* or conditional knockout mutants to challenging conditions like infection *in vivo*, growth in macrophages, growth in metabolic inactive host cells, or during extended extracellular life. These findings show that the endogenous activity of TgFPPS, while low compared to other FPPs (Table S1) is needed under stress or for other functions.

This ability of the parasite to use not only its own metabolites but also to manipulate the host cell metabolism and salvage its products makes it a challenge for drug therapy. However, in the case of isoprenoid metabolism this split reliance may also prove to be an opportunity as it can build on the massive investment made into controlling this pathway pharmacologically in the host. Our work demonstrates that inhibition of the host mevalonate pathway enhances the impact of blocking the parasite isoprenoid pathway and we propose a double hit strategy that combines inhibitors of the parasite enzyme with host isoprenoid pathway inhibitors. We tested combinations of two approved and widely used drugs, zoledronic acid (Zometa) and atorvastatin (Lipitor) and showed synergism in the inhibition of *T. gondii* growth. We demonstrated that impinging on host or parasite isoprenoid synthesis reduces parasite virulence but that blocking both produces stronger effects and affords considerable protection. This strategy could prove even more promising when tested in other parasites. For example our experiments combining fosmidomycin with atorvastatin suggest that atorvastatin may boost the efficacy of fosmidomycin as an antimalarial. This combination may also make it more difficult for the parasite to develop resistance extending the useful life of the drug. Our strategy will benefit from the extensive clinical knowledge on both statins and bisphosphonates and this knowledge will facilitate their use for the treatment of other infections.

## Materials and Methods

### Ethics statement

Mice experiments in this work followed a reviewed and approved protocol by the Institutional Animal Care and Use Committee (IACUC). Animal protocols followed the US Government principles for the Utilization and Care of Vertebrate animals. The protocol was reviewed and approved by the University of Georgia IACUC (Protocol number A2012-3-010).

### Materials

Oligonucleotide primers were obtained from Integrated DNA Technologies (Coralville, IA). Taq DNA polymerase, and restriction enzymes were from Invitrogen or New England Biolabs. Plasmid miniprep and maxiprep and gel kits were from Qiagen Inc. (Chatsworth, CA). IPP, DMAPP, GPP, FPP, GGPP were from Isoprenoids, LC (FL, USA). [4-^14^C] Isopentenyl diphosphate triammonium salt (55.0 mCi/mmol) and [^14^C(U)]-glucose (319 mCi/mmol) were from PerkinElmer Life Sciences. Atorvastatin (Lipitor) was from Pfizer. Zoledronic acid and WC-9 were a gift from Dr. Juan B. Rodriguez, University of Buenos Aires. Fosmidomycin was a gift from Dr. Yongcheng Song (Baylor College of Medicine). All other reagents were analytical grade.

### Cell cultures and transfection of *T. gondii* tachyzoites

Tachyzoites of *T. gondii* RH strain were cultured in human fibroblasts or hTERT cells in Dulbecco's modified Eagle's medium (DMEM) supplemented with 1% fetal bovine serum, 2 mM glutamine, and 1 mM pyruvate, and purified as described before [8]. A basic electroporation protocol was used for transfection. Briefly, 10^7^ recently released parasites and 20 µg of sterilized cosmid (see below) or plasmid DNA were mixed in a 2-mm gap electroporation cuvette. Electroporation was performed using a Genepulser Xcell from BioRad and after 15 min of recovery the parasites were allowed to infect fibroblasts. Stable transfectants were selected with 20 µM chloramphenicol and cloned by limited dilution. For the TgFPPS cDNA complemented cell line, the *Δfpps* parasites were transfected with a TgFPPS cDNA construct [8], then cultured in 15 µM atorvastatin for 4 passages. These parasites were sub-cloned by limited dilution medium containing 5 µM atorvastatin. Subcloned parasites were analyzed by PCR, Southern and western blot. All the clones that survived to atorvastatin selection have the *TgFPPS* cDNA stably integrated. The complemented clone used for the experiments was named *Δfpps-cm*.

The protocol for creating TgFPPS conditional deletion mutants is described in the supporting information (Fig. S2 legend).

### Construction of the *TgFPPS* knockout cosmid

The cosmid PSBLI36TV was obtained from L. David Sibley (Washington University). The knockout cassette from pH3CG was amplified by using the primers (5′-GCGGCCACCGTCCATAATTGCAAAAATGGAGCGGCTGTGTTTCCGTCTCCTCGACTACGGCTTCCATTGGCAAC-3′ and 5′-CTATTTCTGCCGTTT GTGGAGCCTCCCGAGGACGAGGCCGAAGAAGGCCTATACGACTCACTATAGGGCGAATTGG-3′). The TgFPPS gene targeting cosmid construct was obtained by recombineering in *E. coli* EL250 as described previously [14] (Fig. 1A).

### Parasite growth assays

Plaque assays and growth assay of tagged parasites were performed as described before [7]. Plaquing efficiency was measured infecting hTERT monolayers with 1,000 parasites per well and allowing contact with host cells for 30 min. At this point, wells are washed with PBS, fresh media added and parasites allowed to grow for 4–7 days, fixed and stained with crystal violet [18]. Parental and mutant strains of *T. gondii* were transfected with a plasmid containing a tandem tomato RFP gene and red fluorescent parasites were sorted and subcloned by FACS analysis. *Δku80-rfp*, *Δfpps-rfp* and *Δfpps-cm-rfp* cell clones were obtained. Growth competition assays were performed by mixing strains: *Δfpps* parasites with *Δku80-rfp* and *Δfpps-cm-rfp* cell lines at 20∶1 ratio (5% of red cells in the mixture). These parasite mixtures were used to infect fibroblasts or macrophages. 1×10^6^ parasites were inoculated in each passage. Percentage of red cells at each passage was calculated using a standard curve generated by measuring the fluorescence intensity for a fixed number of cells.

### Southern blot analysis

*T. gondii* genomic DNA was digested with SalI, separated in a 0.8% agarose gel, and transferred to a nylon membrane. The DNA probe was generated by PCR with primers (5′-TGACGCGCTGAGCAGTGGTGAGCA-3′ and 5′-AGCCATTTCAACTTCAAACCGCA-3′). The purified PCR product was ^32^P labeled by random priming.

### Western blot analysis

Western blots were done using an affinity purified rabbit polyclonal antibody raised against TgFPPS at 1∶1500 in PBS-T. The secondary antibody was horseradish peroxidase-conjugated goat anti-rabbit and immunoblots were visualized on blue-sensitive x-ray film by using an ECL detection kit.

### ATP measurements

Purified parasites were washed in Ringer (155 mM NaCl, 3 mM KCl, 1 mM MgCl_2_, 3 mM NaH_2_PO_4_-H_2_O, 10 mM Hepes, pH 7.3, 10 mM glucose) and resuspended in Buffer A with glucose (BAG, 116 mM NaCl, 5.4 mM KCl, 0.8 mM MgSO_4_, 5.5 mM D-glucose and 50 mM Hepes, pH 7.2) at 1–5×10^8^ cells/ml. The parasite suspension was incubated at 37°C for 1 hour. The suspension was extracted with perchloric acid as described previously [42]. Briefly, the parasite suspension was centrifuged and resuspended in 100 µl of BAG and mixed with 300 µl of 0.5 M HClO_4_ and incubated in ice for 30 min. The supernatant was neutralized with 0.72 M KOH/0.6 M KHCO_3_ and used immediately or stored at −20C for ATP measurement. The kit A22066 from Invitrogen was used to measure ATP.

### Metabolic labeling and reverse phase thin layer chromatography

Two labeling strategies were used. The first one consisted on labeling both host cells and parasites. Briefly, hTERT-fibroblasts were first infected with fresh tachyzoites and grown in DMEM medium containing ^14^C-glucose until the natural release of parasites. The second strategy consisted of labeling only host cells and infecting afterwards. Host cells were grown in medium containing ^14^C-glucose and before infection the monolayer was thoroughly washed and fresh medium containing glucose was added. For both experiments, released tachyzoites were purified by several filtration steps (8, 5, and 3 µM membranes), to ensure the absence of host cells, and lipids extracted in chloroform/methanol at 4°C overnight. After filtration followed a saponification step (with KOH and ethanol) and the radioactive prenyl products in the mixture were hydrolyzed to the corresponding alcohols with alkaline phosphatase at room temperature, overnight. The resulting alcohols were extracted with hexane and separated on a HP-TLC-RP18 plate using acetone∶H_2_O (10∶1; v/v) as the moving phase. Standard prenyl alcohols were run in parallel and were visualized by iodine vapor. Radioactivity of the products was also measured by autoradiography or phosphorImaginer analysis.

### *In vitro* drug testing

All parasite clones were grown in fibroblasts using similar conditions to those used for the RH strain. For in vitro drug testing, confluent hTERT monolayers in 96-well plates were first prepared with phenol-free medium containing the drugs serially diluted and infected with 4,000 parasites per well. The plates were incubated at 37°C and the fluorescence measured every day. Regression analysis and IC_50_ calculations were performed using SigmaPlot 10.0.

Isobolograms were constructed by plotting the IC_50_ of one drug against the IC_50_ of the other for each of five drug ratios, with a concave curve indicating synergy, a straight line indicating addition, and a convex curve indicating antagonism. For simplified checkboard studies, drugs were mixed in fixed ratios of their respective IC_50_s and dose-response curves generated from serial dilutions carried out in triplicate. Results were expressed as the sums of the fractional inhibitory concentration (sum FIC = IC_50_ of drug A in mixture/IC_50_ of drug A alone)+(IC_50_ of drug B in mixture/IC_50_ of drug B alone), as described by Berenbaum [43]. Sum FIC values indicate the kinds of interactions as follows: <0.5, synergy; 1, addition; >2, antagonism.

### *In vivo* virulence and drug treatment

For *in vivo* infection with *T. gondii*, fresh tachyzoites were harvested, washed with PBS twice, and resuspended in PBS before inoculation. Female Swiss Webster or BALBc mice were injected with 5–20 tachyzoites of the RH strain i.p. in a 200 µl PBS final volume or 10,000–100,000 tachyzoites of the TATi strain in a similar volume. When using low parasite numbers, plaque assays were performed with the parasite suspensions used to inoculate mice to ensure that the number represented the number of viable and infectious parasites.

Because initial *in vivo* experiments with cultured TATi-derived strains gave inconsistent virulence results, we developed a protocol by which we first infected mice with a high dose (10^6^ parasites) of parasites (parental, knock-in and knock outs) and collected the peritoneal fluid (containing tachyzoites) five days p.i. This suspension was used to infect a confluent flask of fibroblasts and allowed to grow and lyse. The supernatant from these flasks containing tachyzoites was collected, centrifuged and parasites resuspended in the appropriate media to prepare aliquots for freezing in liquid nitrogen. For each experiment, one vial was thawed and passed once through tissue culture before used for infection. Tati-derived strains showed a remarkable recovery in virulence with this treatment. We performed titration experiments and determined that 10,000 parasites of Tati-derived strain (no ATc) are lethal to mice 9–10 days p.i.. Results shown in Figs. S3 and 5C were obtained with parasites previously treated as described.

Drugs were dissolved in phosphate-buffered saline (PBS) containing approximately 2% DMSO, at pH 6.8, and were also inoculated i.p. Treatment was initiated 6 hours after infection and administered daily i.p. for 10 days.

## Supporting Information

Figure S1 **Growth in hTert fibroblasts measured with plaque assays.** Upper row shows that mutant parasites can form plaques of the same size as the parental and CM strains. Lower row are from a similar experiment but using old fibroblasts with more than 40 passages. Parasites from each strain were purified and counted. Each well was infected with 150 parasites and cultured for 8 days. Plaques were visualized with gentian violet as in Nair et al. J. Exp. Med. 208:1547–59, 2011.
(TIF)

Figure S2 **Generation of conditional knock-outs for the** ***TgFPPS*** **.** ***A*** **,** Scheme for conditional knockout. The TgFPPS cDNA was cloned into the plasmid pDt7s4H_3_, which was transfected in the previously described Tati (Trans-Activator trap identified) strain (*Meissner et al, Science 298:837–40, 2002*) (*Mazumdar et al, PNAS 103:13,192–97, 2006*). The resulting transfectants (FPPS/FPPSi) were clonally selected with pyrimethamine and they expressed an extra copy of the *TgFPPS gene*, which could be regulated with tetracycline. These cells were used for deleting the endogenous *TgFPPS gene* by transfecting them with the same cosmid described under Materials and Methods (ΔFPPS/FPPSi) following the published protocol (*Brooks et al, Cell Host Microbe. 7: 62–73, 2010*). ***B*** **,** The overexpression of TgFPPS is regulated by Anhydrotetracycline (ATc). A Western blot analysis for both FPPS/FPPSi and ΔFPPS/FPPSi parasites in the presence and absence of ATc is shown. In the presence of ATc there is a decrease in the level of expression of TgFPPS in the FPPS/FPPSi cells and the remaining reaction is probably from the endogenous FPPS. No reaction corresponding to the TgFPPS is observed when ATc is added to the ΔFPPS/FPPSi cells. This result indicates a clear regulation of the expression levels of TgFPPS by ATc. Cells were grown in the presence of ATc (0.5 µg/ml) for four days. ***C*** **,** Southern blot analysis of genomic DNA extracted from the parental Tati (*lane 1*), FPPS/FPPSi (*lane 2*) and ΔFPPS/FPPSi (*lane 3*) parasites and digested with *SalI*. The DNA probe used was the same one described in Figure 1. The DNA from ΔFPPS/FPPSi (*lane 3*) parasites do not contain the endogenous *TgFPPS* gene. FPPS/FPPSi (*lane 2*) parasites show both the endogenous and the extra copy of the *TgFPPS* gene. ***D*** **,** Plaque assays of FPPS/FPPSi and ΔFPPS/FPPSi parasites in human fibroblasts in the presence and absence of ATc. ΔFPPS/FPPSi cells form normal size and number of plaques in fibroblasts even in the absence of both the extra and endogeneous *TgFPPS gene*. ***E*** **,** ΔFPPS/FPPSi parasites pre-incubated with ATc grows slower (or infect less efficiently) when infecting macrophages as host cells. The same number of tachyzoites was used to infect fibroblast and macrophages. All these strains express tandem tomato RFP and the fluorescence was calibrated with a standard curve for fluorescence intensity vs number of parasites. Red fluorescence was measured at day 5. The fluorescence signal was normalized to the fluorescence intensity of the same cells without pre-incubation with ATC.
(TIF)

Figure S3 **Virulence of TgFPPS conditional mutant parasites.** Groups of 10 mice were infected with 10,000 parasites/mouse of FPPS/FPPSi or ΔFPPS/FPPSi tachyzoites. 5 mice from each group received of 0.2 mg/ml anhydrotetracycline (+ATc), or a placebo (−ATc) in their drinking water. The results shown are from 2 independent experiments.
(TIF)

Figure S4 **TgFPPS mutants show a significant loss of the mitochondrial membrane potential.** *Δku80* and *Δfpps* tachyzoites were labeled with JC1 and analyzed by flow cytometry. *Δfpps* parasites show lower % of cells with both high green and red fluorescence intensity. Parasites were collected, washed with phenol red free medium, resuspended in the same medium containing 1.5 µM JC1 for 15 min. Cells were then washed and analyzed by FACS analysis. The detailed protocol is explained in *Brooks et al. Cell Host Microbe* ***7*** *, 62–73, 2010*.
(TIF)

Table S1 **Comparison of the enzymatic properties of TgFPPS with other characterized FPPSs.**
(PDF)
