# Supplementary figures and images for: Toxoplasma gondii Relies on Both Host and Parasite Isoprenoids and Can Be Rendered Sensitive to Atorvastatin
Source: PLoS Pathog. 2013 Oct 17;9(10):e1003665. doi: 10.1371/journal.ppat.1003665 (PMC3798403; doi:10.1371/journal.ppat.1003665)

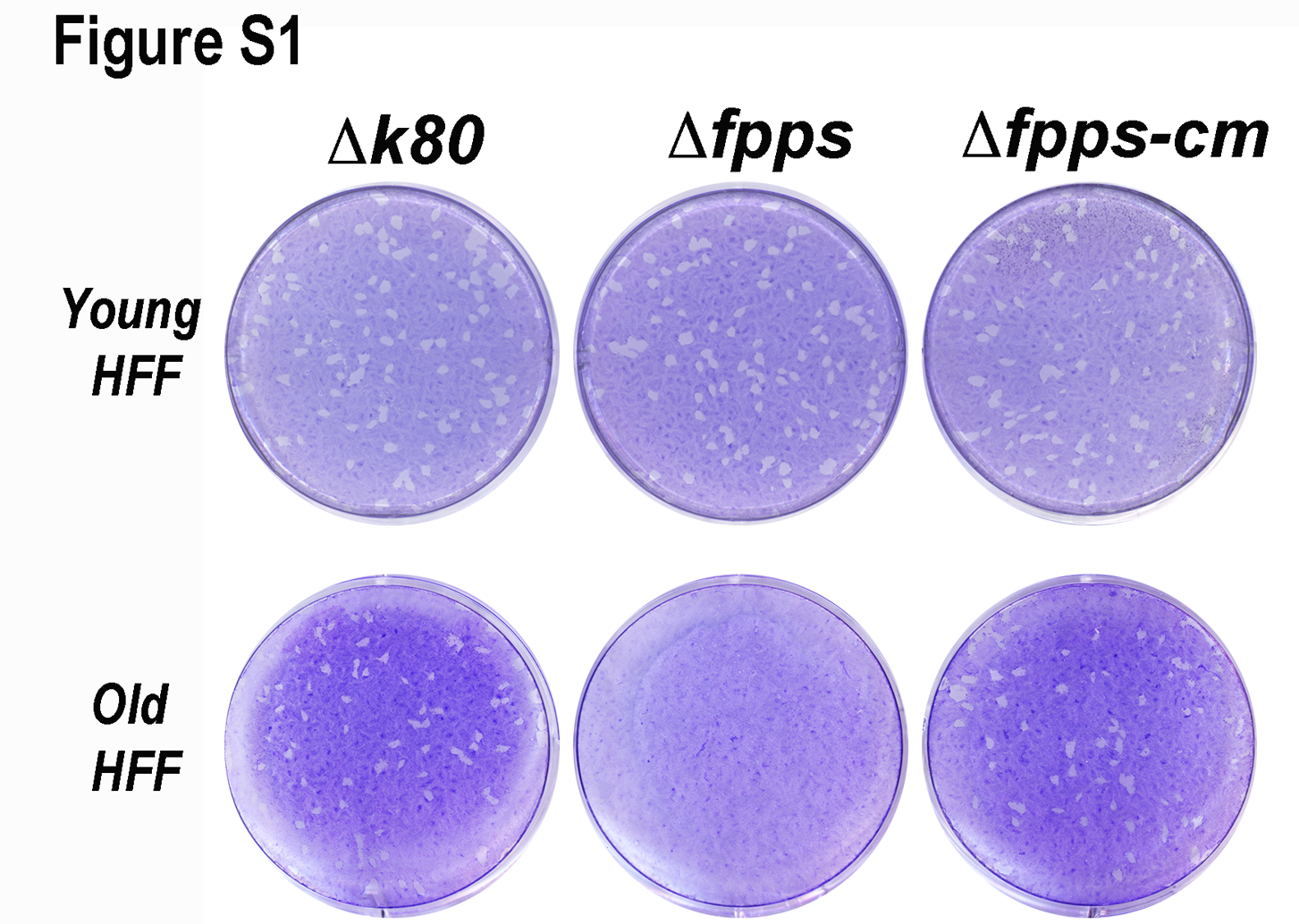

Supplement: Figure S1 — Growth in hTert fibroblasts measured with plaque assays. Upper row shows that mutant parasites can form plaques of the same size as the parental and CM strains. Lower row are from a similar experiment but using old fibroblasts with more than 40 passages. Parasites from each strain were purified and counted. Each well was infected with 150 parasites and cultured for 8 days. Plaques were visualized with gentian violet as in Nair et al. J. Exp. Med. 208:1547–59, 2011. (TIF) [file ppat.1003665.s001.tif]

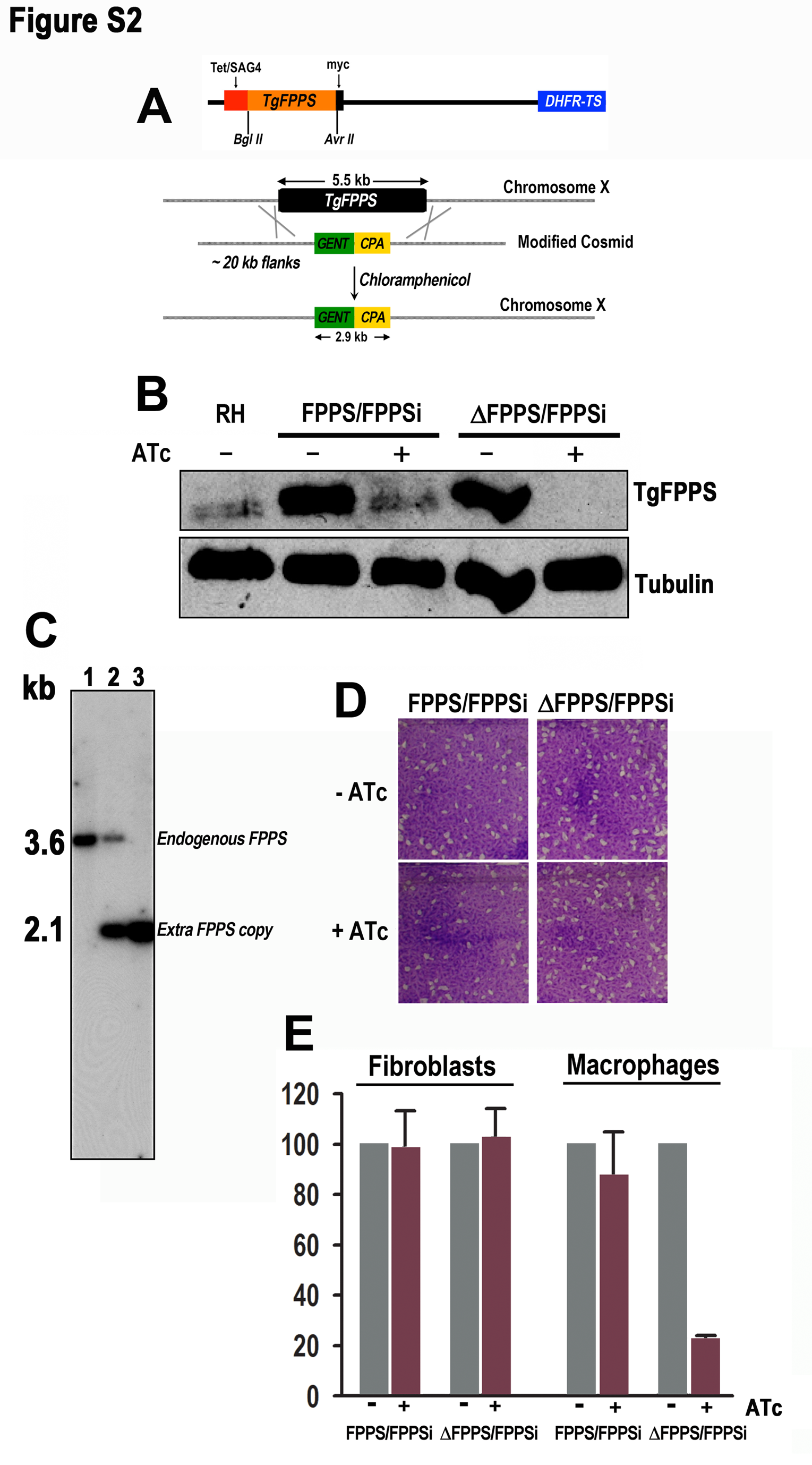

Supplement: Figure S2 — Generation of conditional knock-outs for the TgFPPS . A , Scheme for conditional knockout. The TgFPPS cDNA was cloned into the plasmid pDt7s4H3, which was transfected in the previously described Tati (Trans-Activator trap identified) strain (Meissner et al, Science 298:837–40, 2002) (Mazumdar et al, PNAS 103:13,192–97, 2006). The resulting transfectants (FPPS/FPPSi) were clonally selected with pyrimethamine and they expressed an extra copy of the TgFPPS gene, which could be regulated with tetracycline. These cells were used for deleting the endogenous TgFPPS gene by transfecting them with the same cosmid described under Materials and Methods (ΔFPPS/FPPSi) following the published protocol (Brooks et al, Cell Host Microbe. 7: 62–73, 2010). B , The overexpression of TgFPPS is regulated by Anhydrotetracycline (ATc). A Western blot analysis for both FPPS/FPPSi and ΔFPPS/FPPSi parasites in the presence and absence of ATc is shown. In the presence of ATc there is a decrease in the level of expression of TgFPPS in the FPPS/FPPSi cells and the remaining reaction is probably from the endogenous FPPS. No reaction corresponding to the TgFPPS is observed when ATc is added to the ΔFPPS/FPPSi cells. This result indicates a clear regulation of the expression levels of TgFPPS by ATc. Cells were grown in the presence of ATc (0.5 µg/ml) for four days. C , Southern blot analysis of genomic DNA extracted from the parental Tati (lane 1), FPPS/FPPSi (lane 2) and ΔFPPS/FPPSi (lane 3) parasites and digested with SalI. The DNA probe used was the same one described in Figure 1. The DNA from ΔFPPS/FPPSi (lane 3) parasites do not contain the endogenous TgFPPS gene. FPPS/FPPSi (lane 2) parasites show both the endogenous and the extra copy of the TgFPPS gene. D , Plaque assays of FPPS/FPPSi and ΔFPPS/FPPSi parasites in human fibroblasts in the presence and absence of ATc. ΔFPPS/FPPSi cells form normal size and number of plaques in fibroblasts even in the absence of both the extra and endogeneous [file ppat.1003665.s002.tif]

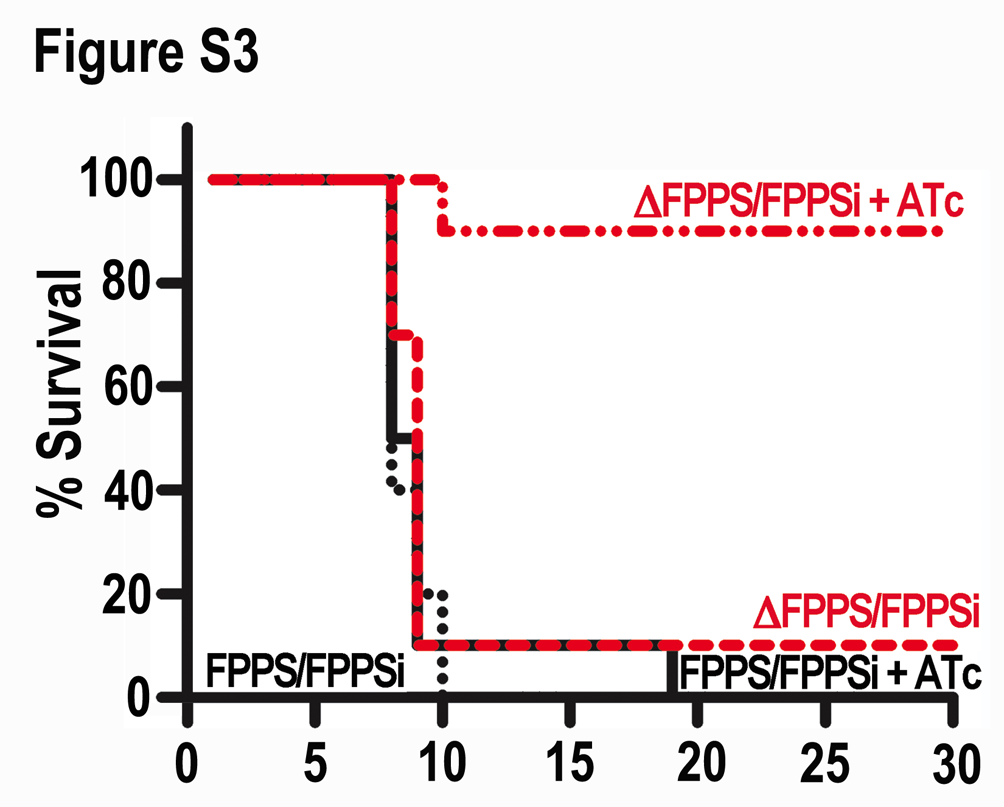

Supplement: Figure S3 — Virulence of TgFPPS conditional mutant parasites. Groups of 10 mice were infected with 10,000 parasites/mouse of FPPS/FPPSi or ΔFPPS/FPPSi tachyzoites. 5 mice from each group received of 0.2 mg/ml anhydrotetracycline (+ATc), or a placebo (−ATc) in their drinking water. The results shown are from 2 independent experiments. (TIF) [file ppat.1003665.s003.tif]

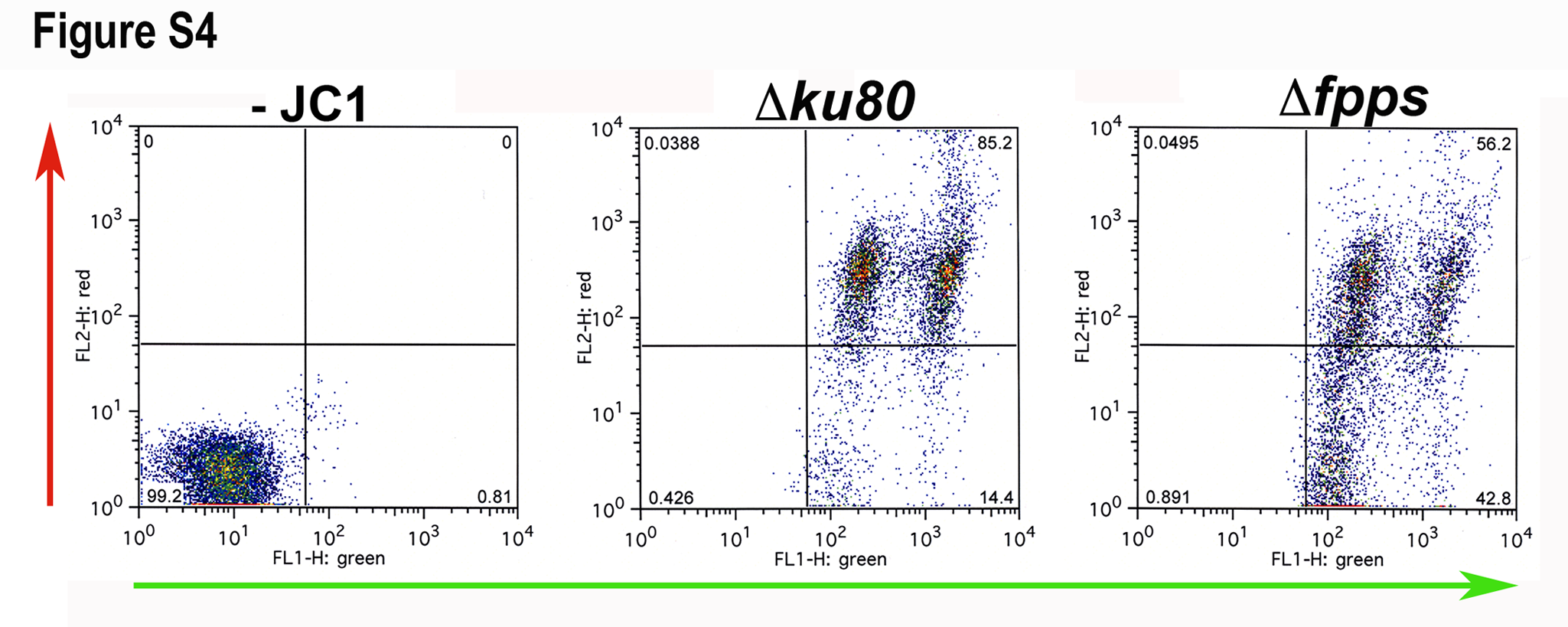

Supplement: Figure S4 — TgFPPS mutants show a significant loss of the mitochondrial membrane potential. Δku80 and Δfpps tachyzoites were labeled with JC1 and analyzed by flow cytometry. Δfpps parasites show lower % of cells with both high green and red fluorescence intensity. Parasites were collected, washed with phenol red free medium, resuspended in the same medium containing 1.5 µM JC1 for 15 min. Cells were then washed and analyzed by FACS analysis. The detailed protocol is explained in Brooks et al. Cell Host Microbe 7 , 62–73, 2010. (TIF) [file ppat.1003665.s004.tif]
