# Supplementary material for: Toxoplasma gondii Relies on Both Host and Parasite Isoprenoids and Can Be Rendered Sensitive to Atorvastatin
Source: PLoS Pathog. 2013 Oct 17;9(10):e1003665. doi: 10.1371/journal.ppat.1003665 (PMC3798403; doi:10.1371/journal.ppat.1003665)
Supplement: Table S1 — Comparison of the enzymatic properties of TgFPPS with other characterized FPPSs. (PDF) [file ppat.1003665.s005.pdf]

Table S1: The activity of the TgFPPS is low as compared to the human and kinetoplastid enzymes.

**Organism enzyme**	**V_{max} (nmol/min/mg)**	**K_m (μM)**
T. gondii	27.1±0.9	1.0±0.1
T. cruzi	214±12	7.48±1.25
L. major	3225±12	13.77±0.3
Human	1080±240	2.07±0.2

The values for V_{max} and K_ms were from the following references:

T. gondii: Ling Y. et al. (2007) *J. Biol. Chem.* 282:30804-16.

T. cruzi: Montalvetti A. et al (2001) *J. Biol. Chem.* 276:33930-7.

L. major: Ortiz-Gómez A. et al (2006) *Euk. Cell.* 5:1057–64.

Human: Ding V.D. et al. (1997) *Biochem. J.* 275:61-65 (V_{max}) and Kavanagh K.L. et al. (2006) *PNAS,* 103:7829-34 (K_m)
